# The Transit Phase of Migration: Circulation of Malaria and Its Multidrug-Resistant Forms in Africa

**DOI:** 10.1371/journal.pmed.1001040

**Published:** 2011-05-31

**Authors:** Caroline Lynch, Cally Roper

**Affiliations:** London School of Hygiene and Tropical Medicine, London, United Kingdom

## Abstract

In the third article in a six-part <I>PLoS Medicine</I> series on Migration & Health, Cally Roper and Caroline Lynch use a case study of migration and anti-malarial drug resistance in Uganda to discuss the specific health risks and policy needs associated with the transit phase of migration.

**Summary Points**

- Movement of people is the means by which human pathogens are dispersed, so providing health care to mobile sectors of the community is vital to disease control interventions.
- Using malaria as a case study, our article examines types of migrant transit and their significance for prevention and treatment of the disease.
- Asymptomatic untreated infections act as reservoirs for malaria transmission. Malaria control programmes need to identify those migrant streams with potential to transport malaria and to target prevention and treatment measures appropriately.
- Transit has also played a role in the dispersal of antimalarial drug resistance, with the international transportation of artemisinin-resistant parasites by human migration being the greatest threat to the antimalarial treatments used in Africa today.
- A geographic framework of human migration at local, national, and international levels is needed so the potential speed and direction of pathogen dispersal can be predicted, and for health policy and services to respond appropriately to the needs of migrants and to threats of pandemic.

**This is one article in a six-part** ***PLoS Medicine*** **series on Migration & Health**.

## Transit Migration

There are many different types of migrants and types of movements and no single commonly agreed definition for transit migration. Papadopoulou [1] describes it as “the stage between emigration and settlement”, while in contrast, the assembly of Inter-Parliamentary Union in Geneva [2] states that “transit migrants are…aliens who stay in the country for some period of time while seeking to migrate permanently to another country”.

Although transit is often defined in terms of international borders, it is highly likely that transit migrants undertake similar patterns of movement within their countries of origin, and within the transit and settlement countries. Within a country people can be moving permanently or multiple times towards urban areas and back home, especially if they are internally displaced people (IDPs). In practice, transitory migration is one component within a broad framework of movement encompassing permanent, temporary, and circulatory migration, all of which can occur in a bidirectional or in a stepwise sequence of moves [3],[4].

This article examines types of transit migration and their significance for the prevention and treatment of malaria in Africa, using a case study of migration in southwest Uganda.

## People Movement and Malaria

*Plasmodium falciparum* malaria—responsible for the most severe malaria—occurs across Africa, although the intensity of transmission varies considerably. Half of Africans live in areas of high endemicity where more than 40% of the population have parasites in their blood [5]; the bulk of these infections are asymptomatic. Transit between different areas exposes people to changing risks of malaria infection, and the burden of infection often falls disproportionately on mobile and migrant sectors of the community [6].

Migrants travelling from low to high transmission areas are at greater risk of acquiring a malarial infection than those travelling in the opposite direction, where also having no acquired immunity means they are much more vulnerable to disease. Evidence from large-scale population resettlement programs in Ethiopia [7], Indonesia, and Brazil [8] show sharp increases in malaria morbidity and mortality across all age groups in migrants from low to high transmission areas.

As such, migration has enormous significance for patterns of malaria infection and disease, and for malaria control. When large groups of people move from high to low transmission areas, the immediate result, as measurable by parasite prevalence, would be an overall increase in transmission [9]. But more common than unidirectional permanent migrations are the regular and cyclical movements of migrants or return migrants to areas of low transmission. In either case, a migrant infected with malaria can serve as a reservoir and seed localised outbreaks or epidemics in those areas, and thus migrants become “active transmitters” of infection in low transmission areas [10].

## Migration, Malaria, and Drug Resistance

International transit of people with malaria played a significant role in the global dispersal of resistance to both chloroquine and sulphadoxine-pyrimethamine (also known as SP or Fansidar), two drugs that were the mainstay of malaria treatment in Africa for 30 years. In both cases, resistance mutations arose in Southeast Asia and were subsequently imported into Africa during the 1970s for chloroquine, and during the 1980s and 1990s for pyrimethamine and sulphadoxine, respectively [11],[12]. While there was a 17-year lag between the appearance of chloroquine resistance in Southeast Asia and its introduction to Africa, once established in Africa the dispersal of resistance has been shown to follow the predictable process of incremental diffusion [12].

**Figure 1:**
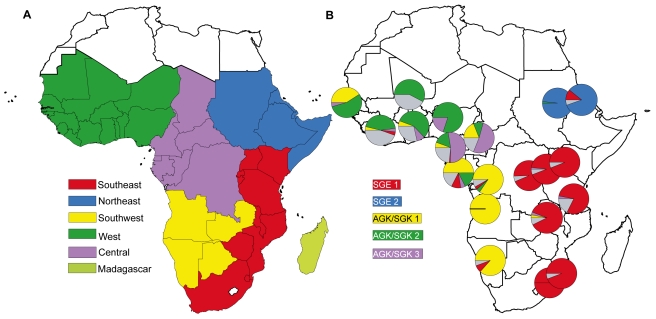
Regions of malaria parasite exchange strongly resemble the distribution of resistance allele lineages. (A) *P. falciparum* migration communities for Africa. The map shows communities connected by comparatively higher levels of malaria migration, with community membership shown by colour (from Tatem and Smith [13]). (B) The distribution of resistance mutant lineages among 20 African *P falciparum* populations (reprinted from Pearce et al. [14]). Most resistance mutations in the *dhps* gene belong to five major lineages, (indicated by the colours shown in the key) and each is derived from a single emergence event. Those which do not belong to the major five are shown in grey.

In 2010, Tatem et al. [13] used permanent migration data derived from national census statistics and measures of malaria endemicity to describe communities of malaria-endemic African countries linked by higher levels of infection movement (see Figure 1A). Their analysis highlights “natural” migration regions where high levels of malaria infection are interchanged. A comparison of malaria migration data (Figure 1A) with the geographic distribution of parasite drug resistance lineages (Figure 1B) shows there is broad correspondence between migration and resistance patterns.

The description of the geographic patterns of drug resistance dispersal comes from an analysis [14] that described five major lineages of sulphadoxine resistance at the *dhps* gene in *P. falciparum*. These emerged during the 1990s when the SP drug was widely used for treatment of malaria. Each lineage occurred via a single mutation event, and has a distinctive geographical distribution that reflects circulation and dispersal from their site of initial emergence [14]. The map in Figure 1b is a snapshot of the distribution of these resistance lineages among 20 African populations sampled during 2000–2007. The degree to which parasite populations share resistance lineages is an indication of the extent of parasite mixing during the 10–15 years they have been in circulation. The major regional distinctions between parasite populations on the African mainland are between populations in the east and west, and between the northern and southern populations in the east and the west.

The similarities between the geographical pattern of resistance mutation dispersal in Africa and the levels of permanent migration between countries support the view that parasite circulation through regionally distinct migration streams was central to the spread of drug resistance mutations. However, permanent migration reported in census data as “place of birth” captures only a fraction of the migration and transit taking place. Furthermore, having been collated at the national level, these data lack the spatial resolution to examine migration and mobility *within* countries. Patterns of human circulation change according to the dictates of war, trade, and transport infrastructure. These maps suggest the existence of networks of high volume migration, whose boundaries are unlikely to be national borders.

## Case Study: Complex Migration Patterns and Drug-Resistant Malaria in Southwest Uganda

**Figure 2:**
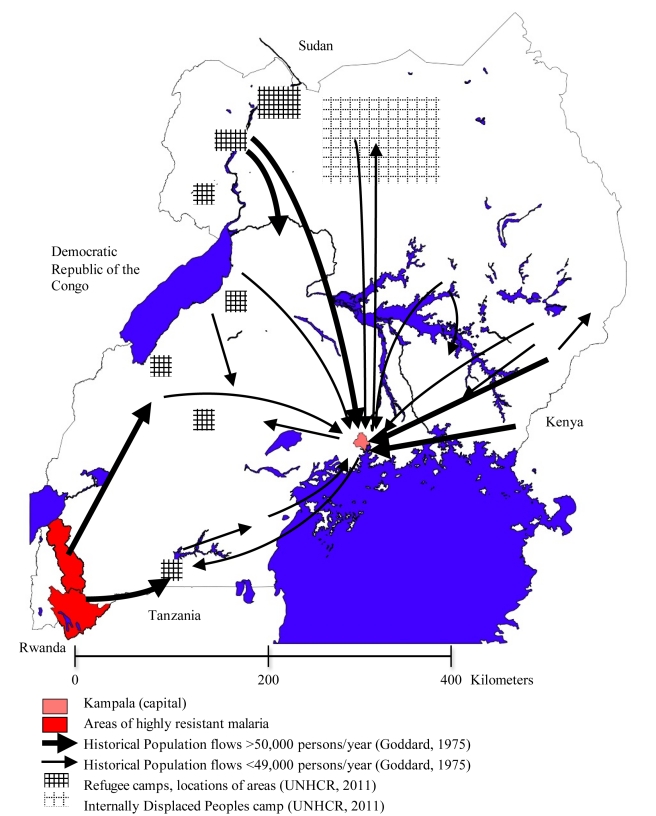
The complex patterns of migration flow in Uganda. The area of highly drug-resistant malaria is shown in red, major population migration flows are shown by arrows, major refugee camps are hatched, and the major urban population in Kampala is indicated in pink. The permanent migration streams are estimated from birthplace data from 1969 population census from Goddard [37].

There has emerged a highly resistant form of *P. falciparum* in the Kabale and Rukungiri districts of southwest Uganda [15] and in bordering areas of Rwanda [16]. The form carries a mutation in the *dhfr* gene, which confers high level resistance to a number of antifolate drugs in general use (including sulfadoxine-pyrimethamine and chlorproguanil-dapsone). To predict the likely path of dispersal of this resistant form of the parasite, we examined the types and frequency of population movements in Uganda. The region of resistance emergence is shown in red in Figure 2.

In 2005, Uganda hosted an international migrant pool of 2.25% of its total population, with a reported net migration of 5,000 people [17]. Internal migration is much higher: nationally, 19.7% of the population of Uganda report to have lived in another village, town, or district for more than 6 months at any one time in the last 5 years [18]. And sub-national or district-level studies have shown that only 18% of heads of households were born in their current areas of residence, and this proportion is higher in rural areas (29%) [19].

Uganda also has a long history of hosting refugees from neighbouring countries. In 2008, the country was home to nearly 250,000 refugees from Sudan, the Democratic Republic of the Congo, and Rwanda in camps containing upwards of 3,000 people each [20], and an additional 915,000 IDPs still remain after being displaced during the ongoing conflict in northern Uganda [21]. The location of refugee and IDP camps is illustrated by shaded regions in Figure 2.

The interregional migrant streams in Figure 2 illustrate the central influence of the main city of Kampala. Urbanization is a major component of permanent migration, although it has progressed more slowly in East Africa than in other parts of the world. In 2002, just over 12% of the total Ugandan population was estimated to live in an urban area [18]. Migration for labour is now accepted as part of a household strategy to improve traditional livelihood [19],[22] and has been relatively well-documented in Uganda. Migrant labour is characterised by circulatory rather than permanent movements, with migrants returning to their areas of origin at the end of a formal contract or wage-paying period [23]. This migration system was disrupted with the economic collapse in the 1970s and 1980s, with reports of reverse migration from major urban areas, such as Kampala, back to rural areas [24]. This reverse flow continued until the mid-1980s [25], when urbanisation began to slowly increase again.

Apart from labour migration, the amount of circulatory migration associated with other activities is difficult to establish. Return migration appears to be frequent with an estimated 10% of urban dwellers making return visits to their home areas of origin annually [26],[27].

### Migration in Southwest Uganda

The southwest of Uganda in particular has always suffered from scarcity of land, as well as erosion and soil degradation [28], which has led to out-migration from the region [29]. More recently, improved roads and increased vehicle numbers, as well as market liberalisation, has led to a further increase in temporary mobility between rural and urban areas [22].

Another result of high population density has been migration and resettlement, both programmed and spontaneous, into neighbouring areas. The first official resettlement schemes took place in the late 1940s, and involved the transfer of approximately 15,000 people from South Kigezi (now Kabale) into North Kigezi (now Rukungiri) [30]. Further resettlement schemes were undertaken to relocate labour from Kabale and Rukungiri to Bunyoro in order to clear 500 square miles of bush as part of a tsetse barrier scheme [31].

While these schemes ended in the 1960s, migration between Kabale, Rukungiri, and the Bunyoro region continued and the original scheme resulted in a strong migration route opening between these areas [32]. Around the same time (mid to late 1950s) in Kabale, people had begun to encroach into the Bwindi forests in the district [33]. Migration data, based on birthplace information taken during the 1969 population census in Uganda, clearly showed a large migration stream from southwestern districts (Kigezi) into the western Toro and Ankole regions as well as into Kampala and its surrounding areas.

These large northward migrant streams from the affected area of southwest Uganda indicate that the highly drug-resistant malaria parasites have the means to spread quickly within Southern Uganda. The maps in Figures 1 and 2 indicate that the future dispersal of resistance has the potential to extend rapidly throughout east and southeast Africa provided that selection in the form of continuing use of SP for treatment is maintained. The relative importance of different types of travel for the dispersal of malaria is difficult to quantify individually, but it is clear from patterns of dispersal of drug resistance that the most significant migration streams carry huge volumes of traffic and that these should be incorporated into thinking about malaria control.

## Geographical Framework of Migration for Malaria Elimination

Programmes for control and eradication of malaria in Africa during the 1950s were undermined by failure to take account of population mobility, and by the difficulties of access to and adequate health care provision for mobile sectors of the population [3]. A geographical framework of malaria dispersal is needed for contemporary national planning and regional coordination of malaria control measures directed towards elimination.

To build such a framework, there is an urgent need for better data on circulation within countries and short-term circulation of populations around porous borders. Countries that have multiple parasite populations, such as the Democratic Republic of the Congo, require detailed surveys of travel behavior across their territory, while countries with large populations of IDPs such as Uganda and Sudan (including Darfur) are also key.

Data on permanent internal migration can be used to describe migration routes and possible migration streams but cannot inform as to the frequency of travel between different places. Permanent migration data from census are informative, but the national scale of the data is not sufficiently high resolution to begin to disentangle the movement of disease within national borders. In particular, permanent migration data will not allow us to capture information on movements between countries with leaky borders, or illegal movement between countries. Some types of circulation in these situations are not captured in surveys because people do not wish to disclose travel across border areas. One example is when IDPs may make a series of return visits to their areas of origin for planting and harvesting but report being present in IDP camps in order to fully avail themselves of food vouchers.

Mapping the dispersal of drug resistance mutations has potential utility in defining regions that encompass significant population mobility and quantifying population connectivity. Such measures summarise the circulation of parasites through all types of migration and have value in this respect, but they are not informative about which migrant sectors of the population should be prioritized for malaria prevention and treatment intervention.

## Need for Migration Data in the Containment of Artemisinin Resistance

As we have noted, migration has historically played a pivotal role in the global dissemination of drug resistance. Therefore, the recent confirmation of resistance to artemisinins on the Cambodia–Thailand border [34] has triggered a concerted effort to contain it. In the policy document “Global Planning Artemisinin Resistance Containment” [35], the World Health Organization identifies three tiers of risk, and implies that migration data are central to their definition. Tier I areas have credible evidence of artemisinin resistance; Tier II areas have significant inflows of people from Tier I; and Tier III areas have no evidence of artemisinin resistance and limited contact with Tier I.

Although most of Africa can be comfortably classified as Tier III, the risk posed by incoming migrants cannot be assessed without knowing the number and final destination of travelers, together with their likelihood of carrying a malaria infection. There is currently no policy for screening travelers to Africa and no interventions to prevent the importation of artemisinin-resistant malaria into Africa. Gathering data on malaria infection rates among incoming travelers should be a priority for African national malaria control programmes.

## Policy Needs and Recommendations

Clearly the current data on transit in Africa are limited, and more migration data are needed. A geographic framework of human migration at local, national, and international levels is particularly essential. It is only through the establishment of such a framework that policy and services can respond appropriately to the needs of migrants and at the same time sustain the gains made in malaria control initiatives. For example, we need to:

- 1. Identify networks of high volume transit and migration within Africa. This should include non-permanent and cyclical transit both within and between countries.
- 2. Apply malaria control and elimination interventions across areas that encompass significant volumes of migration.
- 3. Identify mobile communities and mobile sectors of the population who need to be prioritized in the provision of malaria treatment and prevention measures.
- 4. Provide targeted health care to these communities.

Transit at the inter-continental, national, and local level have all played a role in the dispersal of antimalarial drug resistance in the past. Arguably, the international transportation of artemisinin-resistant parasites by human migration is the greatest threat to the antimalarial treatments used in Africa today. There are currently no policy measures in place to prevent the importation of artemisinin-resistant parasites to Africa. As a result, we also need to:

- 5. Gather data on the volume of migration between areas of confirmed artemisinin resistance and African destinations.
- 6. Establish what the rate of malaria infection among such travelers is and what proportion will travel to areas where transmission can potentially occur. Once the risks of importation are quantified “a speedy, scientifically sound, and coordinated response from affected countries, donors, and international organisations” is needed [36].
- 7. When artemisinin resistance eventually emerges in Africa, apply migration frameworks to guide strategies for the containment or management of resistance through use of alternative treatment combinations.

Molecular markers for resistance to drugs of the past provide insight into the drivers of resistance dispersal. If the rules governing the spread of resistance can be elucidated now, then policy interventions to protect the efficacy of artesimin combination therapies can be designed proactively rather than reactively and the threat of resistance to new treatments managed with foresight.

## Abbreviations

IDP: internally displaced people
